# Contractures of the Hands As a Prenatal Phenotype of *CACNA1A*‐Related Disorder

**DOI:** 10.1002/pd.6827

**Published:** 2025-05-30

**Authors:** Lara Menzies, Alexander Gibbs, Tazeen Ashraf, Clare Beesley, Rowenna Roberts, Natalie J. Chandler

**Affiliations:** ^1^ Department of Clinical Genetics Great Ormond Street Hospital for Children NHS Foundation Trust London UK; ^2^ NHS North Thames Genomic Laboratory Hub Great Ormond Street Hospital for Children NHS Foundation Trust London UK

**Keywords:** *CACNA1A*, contractures, hypotonia, motor delay, saccadic eye movements

**Summary**

- What is already known about this topic?
  ◦ Heterozygous variants in *CACNA1A* are associated with a wide range of variable neurological phenotypes. A prenatal phenotype is not currently recognized for this gene.
- What does this study add?
  ◦ To our knowledge, this is the first reported case of a prenatal neurological phenotype associated with *CACNA1A* related disorder in a fetus presenting with clenched hands, bilateral finger contractures, and adducted thumbs.

## 1 Introduction

A 40 year old multiparous pregnant woman established antenatal care in her first trimester. She and her partner were non‐consanguineous. Maternal history included a previous termination of pregnancy with multiple fetal abnormalities, in whom a molecular diagnosis of trisomy 13 was later confirmed, and a previous healthy male child.

## 2 Fetal Phenotype

Fetal ultrasound scan at 24 weeks gestation in the current pregnancy identified clenched, fist‐shaped hands with apparent bilateral finger contractures and adducted thumbs. There was also a thickened nuchal skin fold of 8.5 mm at 24 weeks, and 13 mm at 25 weeks of gestation (see Table 1a). Finger contractures persisted on subsequent antenatal scans. Mild polyhydramnios was observed on later scans in the third trimester.

**TABLE 1a:** Clinical data.

Case	Parental details	Gestation at diagnosis	Phenotypes (HPO terms)	Obstetric history	Family history	Outcome
1	Maternal: 40 years, White Caucasian	24 weeks	Hand clenching HP:0001188	G4P1: 1 previous pregnancy confirmed diagnosis trisomy 13, 1 early miscarriage, 1 healthy male child	Nil	Planned C section; livebirth
	Paternal: 47 years, Black Caribbean		Congenital finger flexion contractures HP:0005879			
			Adducted thumb HP:0001181			
			Thickened nuchal skin fold HP:0000474			
			Polyhydramnios HP:0001561			

## 3 Diagnostic Method

Prenatal QFPCR and microarray on DNA extracted from uncultured amniotic fluid identified an XX (female) chromosome complement with no evidence of any copy number variants of likely clinical significance regarding the scan findings. Antenatal trio exome sequencing and analysis using a fetal anomaly panel were carried out as previously described [1]. This analysis did not detect any variants that could be considered significant with respect to the ultrasound scan findings. Postnatal trio genome sequencing was performed by the Genomics England laboratory pathway and analyzed using a large pediatric disorder panel [2].

## 4 Pregnancy Outcomes and Neonatal Findings

The mother had no medical problems and reported normal fetal movements throughout the pregnancy. The neonate was born at 37 weeks of gestation by elective Caesarean section. She had a birthweight of 3.6 kg and was born in good condition. No resuscitation or intensive care admission was required. Bilateral contractures of the third, fourth and fifth digits were noted with adducted thumbs. These improved somewhat with physiotherapy over the first months of life. Clinical Genetics review at 9 months of age noted motor developmental delay, hypotonia and brisk reflexes with a general paucity of movement. Hands were still held with adducted thumbs. The child could roll but was not sitting independently. Dystonic posturing was noted in the limbs. A saccadic eye movement disorder was diagnosed with slightly reduced visual evoked potentials (VEP). Eye structure was normal. MRI of the brain noted benign enlargement of the CSF spaces and no other findings. Creatine kinase and baseline metabolic tests were normal.

## 5 Diagnostic Results and Interpretation

Postnatal microarray, Prader‐Willi methylation testing, *DMPK* analysis for myotonic dystrophy and Spinal Muscular Atrophy genetic testing were all unremarkable. Postnatal trio genome sequencing identified a *de novo* heterozygous variant in *CACNA1A* c.1036_1038del p.(Ile346del) (see Table 1b). Heterozygous variants in *CACNA1A* are associated with multiple neurodevelopmental phenotypes including developmental and epileptic encephalopathy (#617106), episodic ataxia type 2 (#108500) and migraine, familial hemiplegic with progressive cerebellar ataxia (#141500). The variant was absent from the gnomADv4.1.0 population database (PM2_moderate) and resulted in a protein length change due to an in‐frame single amino acid deletion in a non‐repeat region (PM4_supporting). The variant was *de novo* in this patient and was also present in another case from the Genomics England cohort with an overlapping matching phenotype (PS2_strong; PS4_moderate). The variant was therefore classified as likely pathogenic [3]. The postnatal clinical findings were consistent with previously reported cases of *CACNA1A*‐related disorder, including the limb contractures [4].

**TABLE 1b:** Genetics findings.

Procedure (gest age)	Direct/culture?	Performed test	Secondary confirmatory test	Gene (name;REfSEQ)	Known disease (OMIM)	Variant	ACMG Classification*	Criteria applied	Inheritance and zygosity	Interpretation
25+6	Direct amniocytes	Trio exome. Fetal anomalies panel applied	None	N/A	N/A	N/A	N/A	N/A	N/A	N/A
Postnatal	Direct blood	Trio genome. Pediatrics disorders panel.	Sanger sequencing	*CACNA1A* (NM_001127221.2)	Developmental and epileptic encephalopathy 42 (MIM 617106), episodic ataxia, type 2 (MIM 108500), and migraine, familial hemiplegic, 1 and migraine, familial hemiplegic, 1, with progressive cerebellar ataxia (MIM 141500).	c.1036_1038del p.(Ile346del) heterozygous	4	PM2_moderate; PS4_moderate; PM4_supporting; PS2_strong	AD, *de novo*	Consistent with diagnosis

^*^ variant classification and criteria using the United Kingdom Association of Clinical Genetics society guidelines 2020.

## 6 Discussion

This case describes an infant with prenatal contractures of both hands, raised nuchal translucency and polyhydramnios, who developed motor delay, hypotonia, dystonia, and a saccadic eye disorder after birth, consistent with a *CACNA1A*‐related disorder. There has been a case report of polyhydramnios identified on antenatal ultrasound in a neonate that would go on to be diagnosed with a *CACNA1A* disorder after birth [5]. However, no prior evidence could be found for prenatal contractures being associated with *CACNA1A* variants. This case suggests that prenatal limb contractures is a rare but early detectable manifestation of *CACNA1A*‐related conditions, particularly when identified in conjunction with polyhydramnios.

## Ethics Statement

Written parental consent was obtained to share the details of this case.

## Conflicts of Interest

LM has received personal fees for *ad hoc* consultancy work for Mendelian Ltd, a digital rare disease health company. All other authors have no conflict of interest to declare.

## Data Availability

The data that supports the findings of this report are available from the authors upon a reasonable request.

## References

[1] N. J. Chandler , E. Scotchman , R. Mellis , V. Ramachandran , R. Roberts , and L. S. Chitty , “Lessons Learnt From Prenatal Exome Sequencing,” Prenatal Diagnosis 42, no. 7 (2022): 831–844, 10.1002/pd.6165. 35506549 PMC9325487

[2] Genomics England PanelApp: Paediatric Disorders Panel v60.9, (2024), https://panelapp.genomicsengland.co.uk/panels/486/.

[3] S. Ellard , E. Baple , A. Callaway , et al., ACGS Best Practice Guidelines for Variant Classification in Rare Disease 2020 2020, https://www.acgs.uk.com/media/11631/uk‐practice‐guidelines‐for‐variant‐classification‐v4‐01‐2020.pdf.

[4] Epi4K Consortium , “De Novo Mutations in *SLC1A2* and *CACNA1A* Are Important Causes of Epileptic Encephalopathies,” American Journal of Human Genetics 99, no. 2 (2016): 287–298, 10.1016/j.ajhg.2016.06.003. 27476654 PMC4974067

[5] M. Ozdil , A. Eroglu , and H. B. Gerik‐Celebi , “A Novel *CACNA1A* Mutation in a Neonate With Severe Encephalopathy at Birth,” Acta Neurologica Belgica 124, no. 2 (2024): 705–708, 10.1007/s13760-023-02453-1. 38079102

